# Supplementary material for: Resolving the full spectrum of human genome variation using Linked-Reads
Source: Genome Res. 2019 Apr;29(4):635–45. doi: 10.1101/gr.234443.118 (PMC6442396; doi:10.1101/gr.234443.118)
Supplement: Supplemental Material [file supp_gr.234443.118_Supplemental_longranger-2.2.zip › longranger-2.2.tar.bz2!/lr2.2-public/lib/stan/cmdstan/stan/doc/api/html/agrad_2rev_2matrix_2multiply_8hpp_source.html]

Stan: src/stan/agrad/rev/matrix/multiply.hpp Source File


|  |  |
| --- | --- |
|  | Stan  2.5.0  probability, sampling & optimization |


- Main Page
- Related Pages
- Namespaces
- Classes
- Files

- File List
- File Members

All Classes Namespaces Files Functions Variables Typedefs Enumerations Enumerator Friends Macros Pages

- src
- stan
- agrad
- rev
- matrix

multiply.hpp

Go to the documentation of this file.

1 #ifndef STAN\_\_AGRAD\_\_REV\_\_MATRIX\_\_MULTIPLY\_HPP

2 #define STAN\_\_AGRAD\_\_REV\_\_MATRIX\_\_MULTIPLY\_HPP

3

4 #include <stan/agrad/rev/var.hpp>

5 #include <stan/agrad/rev/matrix/Eigen\_NumTraits.hpp>

6 #include <stan/agrad/rev/matrix/typedefs.hpp>

7 #include <stan/math/matrix/Eigen.hpp>

8 #include <stan/math/matrix/typedefs.hpp>

9 #include <stan/math/error\_handling/matrix/check\_multiplicable.hpp>

10 #include <stan/agrad/rev/matrix/to\_var.hpp>

11 #include <stan/agrad/rev/matrix/dot\_product.hpp>

12 #include <stan/agrad/rev/operators/operator\_multiplication.hpp>

13 #include <boost/utility/enable\_if.hpp>

14 #include <boost/type\_traits.hpp>

15 #include <boost/math/tools/promotion.hpp>

16

17 namespace stan {

18  namespace agrad {

19

26  template <typename T1, typename T2>

27  inline typename

28  boost::enable\_if\_c< (boost::is\_scalar<T1>::value || boost::is\_same<T1,var>::value) &&

29  (boost::is\_scalar<T2>::value || boost::is\_same<T2,var>::value),

30  typename boost::math::tools::promote\_args<T1,T2>::type>::type

31  multiply(const T1& v, const T2& c) {

32  return v \* c;

33  }

34

41  template<typename T1,typename T2,int R2,int C2>

42  inline Eigen::Matrix<var,R2,C2> multiply(const T1& c,

43  const Eigen::Matrix<T2, R2, C2>& m) {

44  // FIXME: pull out to eliminate overpromotion of one side

45  // move to matrix.hpp w. promotion?

46  return to\_var(m) \* to\_var(c);

47  }

48

55  template<typename T1,int R1,int C1,typename T2>

56  inline Eigen::Matrix<var,R1,C1> multiply(const Eigen::Matrix<T1, R1, C1>& m,

57  const T2& c) {

58  return to\_var(m) \* to\_var(c);

59  }

60

71  template<typename T1,int R1,int C1,typename T2,int R2,int C2>

72  inline typename

73  boost::enable\_if\_c< boost::is\_same<T1,var>::value ||

74  boost::is\_same<T2,var>::value,

75  Eigen::Matrix<var,R1,C2> >::type

76  multiply(const Eigen::Matrix<T1,R1,C1>& m1,

77  const Eigen::Matrix<T2,R2,C2>& m2) {

78  stan::math::check\_multiplicable("multiply(%1%)",m1,"m1",

79  m2,"m2",(double\*)0);

80  Eigen::Matrix<var,R1,C2> result(m1.rows(),m2.cols());

81  for (int i = 0; i < m1.rows(); i++) {

82  typename Eigen::Matrix<T1,R1,C1>::ConstRowXpr crow(m1.row(i));

83  for (int j = 0; j < m2.cols(); j++) {

84  typename Eigen::Matrix<T2,R2,C2>::ConstColXpr ccol(m2.col(j));

85  if (j == 0) {

86  if (i == 0) {

87  result(i,j) = var(new dot\_product\_vari<T1,T2>(crow,ccol));

88  }

89  else {

90  dot\_product\_vari<T1,T2> \*v2 = static\_cast<dot\_product\_vari<T1,T2>\*>(result(0,j).vi\_);

91  result(i,j) = var(new dot\_product\_vari<T1,T2>(crow,ccol,NULL,v2));

92  }

93  }

94  else {

95  if (i == 0) {

96  dot\_product\_vari<T1,T2> \*v1 = static\_cast<dot\_product\_vari<T1,T2>\*>(result(i,0).vi\_);

97  result(i,j) = var(new dot\_product\_vari<T1,T2>(crow,ccol,v1,NULL));

98  }

99  else /\* if (i != 0 && j != 0) \*/ {

100  dot\_product\_vari<T1,T2> \*v1 = static\_cast<dot\_product\_vari<T1,T2>\*>(result(i,0).vi\_);

101  dot\_product\_vari<T1,T2> \*v2 = static\_cast<dot\_product\_vari<T1,T2>\*>(result(0,j).vi\_);

102  result(i,j) = var(new dot\_product\_vari<T1,T2>(crow,ccol,v1,v2));

103  }

104  }

105  }

106  }

107  return result;

108  }

109

119  template <typename T1,int C1,typename T2,int R2>

120  inline typename

121  boost::enable\_if\_c< boost::is\_same<T1,var>::value ||

122  boost::is\_same<T2,var>::value, var >::type

123  multiply(const Eigen::Matrix<T1, 1, C1>& rv,

124  const Eigen::Matrix<T2, R2, 1>& v) {

125  if (rv.size() != v.size())

126  throw std::domain\_error("row vector and vector must be same length in multiply");

127  return dot\_product(rv, v);

128  }

129

130  }

131 }

132 #endif

check\_multiplicable.hpp

typedefs.hpp

stan::agrad::to\_var

var to\_var(const double &x)

Converts argument to an automatic differentiation variable.

**Definition:** to\_var.hpp:21

typedefs.hpp

Eigen\_NumTraits.hpp

stan::agrad::multiply

Eigen::Matrix< fvar< T >, R1, C1 > multiply(const Eigen::Matrix< fvar< T >, R1, C1 > &m, const fvar< T > &c)

**Definition:** multiply.hpp:21

to\_var.hpp

stan::math::check\_multiplicable

bool check\_multiplicable(const char \*function, const T1 &y1, const char \*name1, const T2 &y2, const char \*name2, T\_result \*result)

**Definition:** check\_multiplicable.hpp:15

dot\_product.hpp

stan::agrad::dot\_product

fvar< T > dot\_product(const Eigen::Matrix< fvar< T >, R1, C1 > &v1, const Eigen::Matrix< fvar< T >, R2, C2 > &v2)

**Definition:** dot\_product.hpp:20

operator\_multiplication.hpp

var.hpp

Eigen.hpp

stan::agrad::var

Independent (input) and dependent (output) variables for gradients.

**Definition:** var.hpp:27


---

[ Stan Home Page ]

*© 2011–2014,
Stan Development Team.*
